# TREM-2 serves as a negative immune regulator through Syk pathway in an IL-10 dependent manner in lung cancer

**DOI:** 10.18632/oncotarget.8813

**Published:** 2016-04-18

**Authors:** Yinan Yao, Hequan Li, Junjun Chen, Weiyi Xu, Guangdie Yang, Zhang Bao, Dajing Xia, Guohua Lu, Shuwen Hu, Jianying Zhou

**Affiliations:** ^1^ Department of Respiratory Diseases, First Affiliated Hospital of Zhejiang University, Hangzhou, China; ^2^ Department of Clinical Laboratory, First Affiliated Hospital of Zhejiang University, Hangzhou, China; ^3^ School of Public Health, Zhejiang University, Institute of Immunology, Zhejiang University, Hangzhou, China; ^4^ Institute of Immunology, Zhejiang University, Hangzhou, China

**Keywords:** TREM-2, lung cancer, IL-10, Syk, immunoregulation

## Abstract

During infection, triggering receptor expressed on myeloid cells-2 (TREM-2) restrains dendritic cells (DCs) and macrophages (MΦs) phagocytosis, as well as reduces pro-inflammatory cytokines release through DNAX-activation protein 12 (DAP12) signaling. However, the role of TREM-2 signaling in cancer has never been elucidated. In the current study, we found that TREM-2 was up-regulated on peripheral blood monocytes in tumor-bearing host. More TREM-2^+^DCs were detected in the lung of 3LL tumor-bearing mice. On the other hand, the level of TREM-2 on pulmonary MΦs positively correlated with the pathological staging of lung cancer. However, surgical or chemotherapeutic reduction of tumor burden led to the obvious decline of TREM-2. In vitro, TREM-2 expression of bone marrow (BM)-derived DCs and MΦs was induced by conditional medium (CM) containing the supernatant of 3LL cells. TREM-2^+^DCs from CM and/or tumor-bearing mice held altered phenotypes (CD80^Low^CD86^Low^MHCII^Low^) and impaired functions, such as, reduced interleukin (IL)-12 secretion, increased IL-10 production, and weakened ovalbumin (OVA)-endocytic capacity; also developed potent inhibitory effect on T cell proliferation that could be partially reversed by TREM-2 blockage. Moreover, spleen tyrosine kinase (Syk) inhibitor restrained IL-10 production of TREM-2^+^DC. Remarkably, IL-10 neutralizing antibody and Syk inhibitor both lowered the suppressive potential of TREM-2^+^DCs in T cell proliferation. Also, adoptive transfer of this TREM-2^+^DCs accelerated the tumor growth rather than jeopardized survival in lung cancer-bearing mice. In conclusion, these results indicate that TREM-2 might act as a negative immuno-regulatory molecule through Syk pathway in an IL-10 dependent manner and partially predicts prognosis in lung cancer patients.

## INTRODUCTION

Lung cancer is the most commonly diagnosed cancer as well as the leading cause of cancer death worldwide, presenting a rapid increase of incidence and stubbornly high mortality [1]. Over a period of time, several sophisticated vaccines for active-specific stimulation of the host's own immune system have been developed for lung cancer [2], but frustratingly with a limited efficacy mainly attributable to tumor-specific immunotolerance [3]. More recent evidences have argued for a negative regulatory mechanism that serve to impede the ongoing immune responses, which give a new hope for cancer therapy. Ipilimumab, as one of the human anti-Cytotoxic T Lymphocyte Antigen (CTLA-4) monoclonal antibodies (mAb) applied in the first-line treatment of metastatic non-small lung cancer cell combined with chemotherapy, showed an improvement of survival in comparison with chemotherapy alone, without major added toxicity [4]. Further understanding of negative regulation and discovery of new targets are necessary to represent an attractive immune-therapeutic approach for the treatment of cancer.

TREM-2 is a member of the innate immune receptor belonging to the immunoglobulin superfamily, primarily expressed on the cell surface of the monocyte-macrophage lineage including MΦs, DCs, microglia, and osteoclasts [5, 6]. TREM-2 associates with DAP12 activates the downstream signaling through immuno-receptor tyrosine-based activation motif (ITAM) domain followed by the the recruitment of Syk and launch of signaling cascades [7, 8]. Current evidence proved that TREM-2-deficient BM-derived DCs produced increased inflammatory cytokine and type I interferon (IFN) in response to Toll-like receptor (TLR) agonists, and were more efficient at inducing antigen-specific T-cell proliferation [9]. Tumor necrosis factor (TNF)-α and IL-6 levels were higher in TREM-2 deficient BM-derived MΦs in response to TLR ligands [10]. These studies indicated that TREM-2 negatively regulated TLR responses in DCs and MΦs during inflammation, which led us to speculate that TREM-2 may exert a similar role in the antitumor immunity.

In the current study, we found that TREM-2 expression was up-regulated in the tumor-bearing host while reduction of tumor burden led to the obvious decline of TREM-2. Moreover, it could be induced by CM on BM-derived DCs and MΦs. TREM-2^+^DCs not only presented impaired phenotypes and reduced OVA-endocytic capacity but also performed a more potent inhibitory effect on T cells proliferation. Syk was responsible for TREM-2-mediated inhibitory signaling in lung cancer while Syk inhibitor restrained the IL-10 production of TREM-2^+^DC. Hence, we suggest that TREM-2 might act as a negative immune regulator in lung cancer.

## RESULTS

### TREM-2 expression was up-regulated in the tumor-bearing host

**Figure 1:**
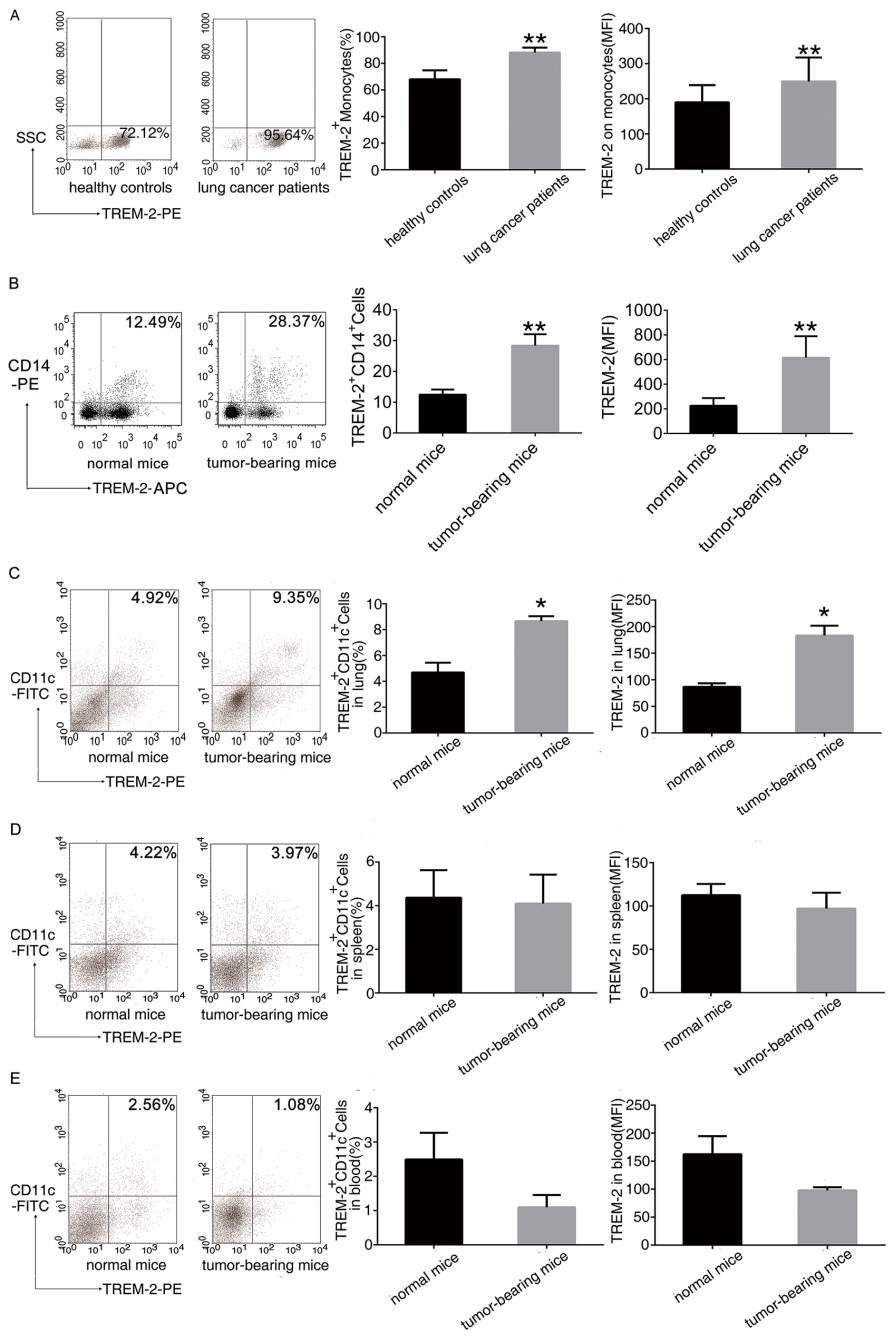
Increase of TREM-2 expression in the tumor-bearing host **A.** Leukocytes isolated from peripheral blood of 52 patients with lung cancer and 41 healthy controls were analyzed by flow cytometry. The percentage of TREM-2^+^monocytes and MFI of TREM-2 expression were shown. **B.** The percentage of TREM-2^+^monocytes and MFI of TREM-2 expression were shown in the blood of normal and tumor bearing- mice. **C-E.** Cells isolated from the lung, spleen, and blood of tumor-bearing mice and normal mice were double-stained with FITC-conjugated CD11c and PE-conjugated TREM-2 and were analyzed by flow cytometry. The percentage of TREM-2^+^ CD11c^+^ cells and MFI of TREM-2 expression of the lung, spleen and blood were shown. Results were presented as mean ± SEM. Numbers indicated are the average values of three independent experiments (n = 6/group). **p < 0.01 compared with healthy controls or normal mice; *p < 0.05 compared with normal mice.

We found that the number of TREM-2^+^monocytes in peripheral blood and the mean fluorescence intensity (MFI) of its expression significantly increased in the lung cancer patients and tumor-bearing mice compared to that in healthy controls (Figure 1A, 1B).

Intriguingly, similar results were observed in an orthotopic lung cancer model. TREM-2 levels of CD11c^+^DCs from lung, spleen and blood were detected by FACS. The number of TREM-2^+^CD11c^+^DCs and the MFI of TREM-2 expression on DCs from lung increased after tumor bearing (Figure 1C) while these two indexes of DCs had no alteration in the spleen and blood of tumor-bearing mice compared to that of normal mice (Figure 1D, 1E).

### Level of TREM-2 on pulmonary MΦs is related to the pathological staging of lung cancer

**Figure 2:**
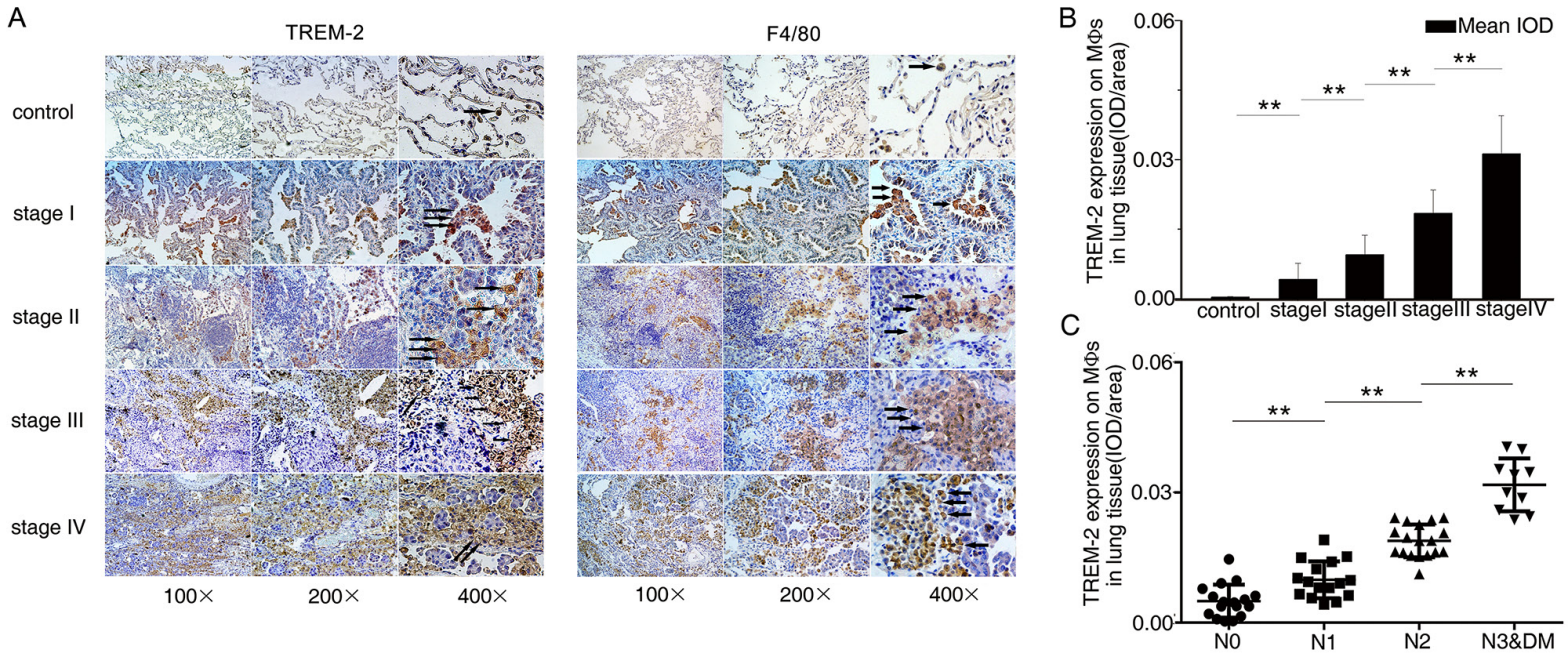
The level of TREM-2 on pulmonary MΦs positively correlated with pathological staging and lymph nodes metastasis of lung cancer **A.** Adjacent lung tissues sections from each group were stained with TREM-2 and F4/80, respectively. Representative photomicrographs were shown (Arrows in A showed TREM-2^+^ MΦs). **B.** The intensity of staining of TREM-2 was quantified using mean integrated optical density (IOD) (mean IOD = sum IOD/sum area). **C.** The degree of lymph nodes metastasis was according to 7th TNM classification. The results represent the mean ± SEM. n: controls, 21; stage I, 15; stage II, 15; stage III, 15; stage IV, 11; **p < 0.01 compared with the previous group. DM: distant metastasis.

We discovered that compared to benign diseases, TREM-2 levels on MΦs were increased in lung cancer patients. Moreover, TREM-2 levels of MΦs around the tumor cells had a positive correlation with TNM stage (Figure 2A, 2B).

We also investigated the effect of TREM-2 on lymph node metastasis among the surgical specimens and observed that TREM-2 expression of MΦs was positively correlated with the degree of lymph nodes metastasis (Figure 2C).

### Reduction of tumor burden leads to obvious decline of TREM-2 on the peripheral blood monocytes

**Figure 3:**
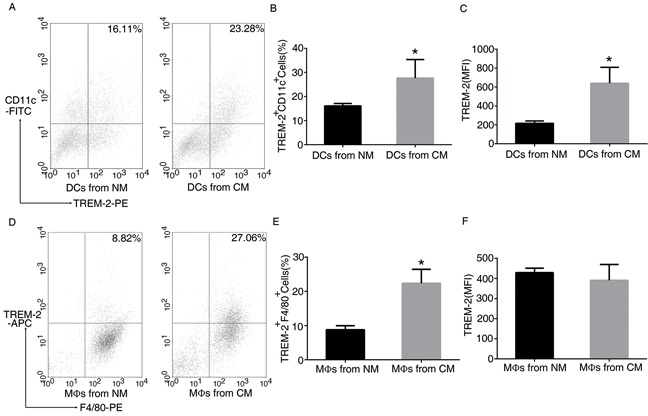
Reduction of tumor burden, by operation or chemotherapy, led to the obvious decline of TREM-2 expression Blood samples were collected from 20 patients with lung cancer before and after chemotherapy (at the beginning of cycle 3) in the department of respiratory medicine (7 patients were squamous cell carcinomas and 13 patients were adenocarcinomas), and 15 patients with lung cancer before and after operation (after one month) in the department of thoracic surgery (5 patients were squamous cell carcinomas and 10 patients were adenocarcinomas). The patients who received chemotherapy were divided into two group according to the therapeutic response: Group1 was including the patients with SD and PD (n=10) while Group2 included the patients with PR (n=10). The last two columns contained the patients with lung cancer before and after the operation. The expression of TREM-2 on monocytes detected by FACS was down-regulated dramatically in patients with PR after chemotherapy and in those who received surgical resection while it was unchanged or even elevated in patients with SD and PD. *p < 0.05 compared with that before chemotherapy; # *p* < 0.05 compared with that before operation.

To further identify the dynamic alterations of TREM-2 in the lung cancer, we compared its levels on the peripheral blood monocytes from the patients before and after chemotherapy or surgery, respectively. The expression of TREM-2 was down-regulated dramatically in both the groups of patients, those who presented partial response (PR) to chemotherapy and those who underwent surgery (Figure 3).

### TREM-2 expression of peripheral blood monocytes may reflect the effectiveness of chemotherapy

Upon assessing the chemotherapeutic responses, we divided the patients with lung cancer into two groups: Group1 was included the patients of stable and progressive disease (SD, PD) while Group2 was included the patients of PR. Surprisingly, we found that the expression of TREM-2 was markedly decreased in the patients with PR, while that was unchanged or even elevated in the patients with SD and PD (Figure 3), suggesting that TREM-2 may be a valuable index for estimating the efficacy of lung cancer chemotherapy.

### TREM-2 on DCs and MΦs can be induced by CM containing the supernatant of 3LL cells

**Figure 4:**
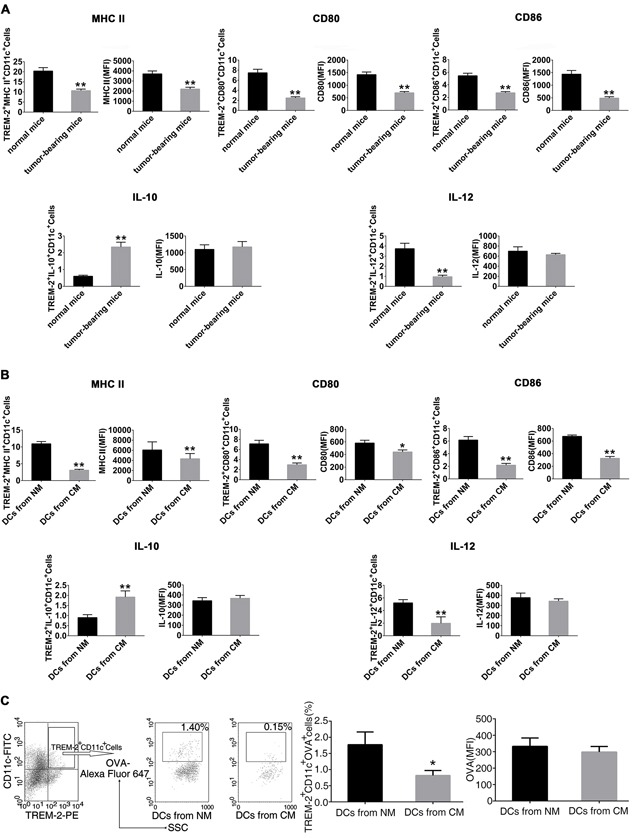
TREM-2 on bone marrow-derived DCs and MΦs was induced by conditional medium (CM) BM-derived DCs and BM-derived MΦs were isolated from C57BL/6J mice. During culture, we prepared CM containing supernatant of 3LL cells (medium: supernatant = 4:1) mimicking tumor microenvironment for DC and MΦ derivation, which was added to the DC medium from day 3 to day 9, and to the MΦ medium from day 3 to day 7. The cells were then collected and double-stained with CD11c-FITC, TREM-2-PE for DCs **A, B, C.** and F4/80-PE. TREM-2-APC for MΦs **D, E, F.** was analyzed by FACS assay. Mean ± SEM from triplicate wells of one representative experiment out of four was shown. *p < 0.05 compared with NM.

In order to determine whether the tumor mediated the induction of TREM-2 in vitro, we prepared CM for DC and MΦ derivation. Interestingly, more TREM-2^+^DCs and TREM-2^+^MΦs were harvested from such medium than that from the normal medium (NM) (Figure 4), indicating that tumor microenvironment could induce TREM-2.

### Cancer-elicited TREM-2^+^DCs had impaired phenotypes and functions

Here we focused on DCs to investigate the effect of TREM-2 during tumor immunoregulation. We noted that TREM-2^+^DCs had more inhibitory characteristics both in vivo and in vitro.

**Figure 5:**
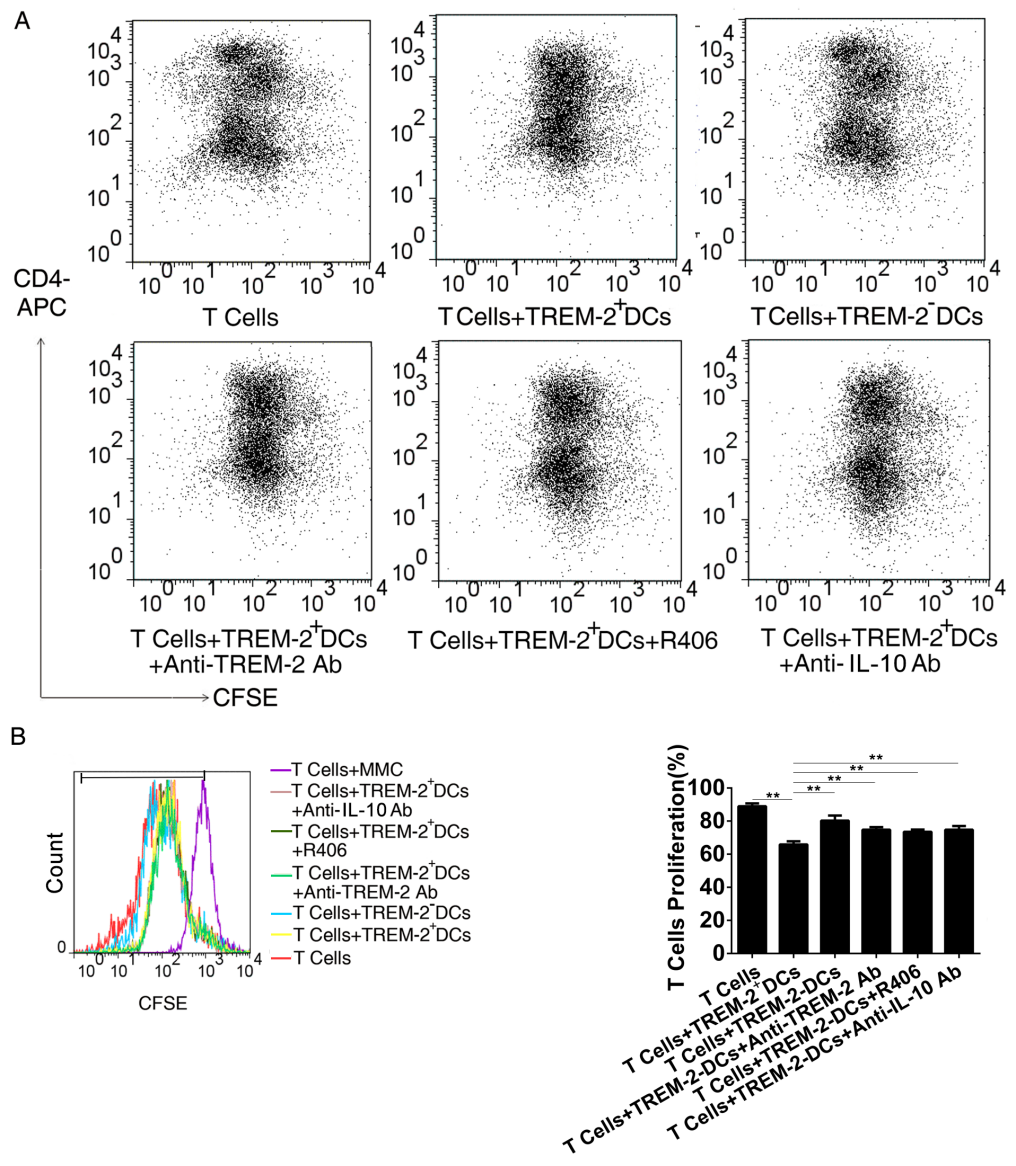
Cancer-elicited TREM-2^+^DCs had impaired phenotypes and functions **A.** CD11c^+^ lung cells were isolated from tumor-bearing and normal mice by magnetic activated cell sorting (MACS); **B.** DCs were harvested from NM and CM, and CD11c^+^ population was enriched by MACS, then double-stained with TREM-2- PE, and PerCP-eFluor-MHCII I-A/I-E or PerCP-Cy5.5-CD80 or PerCP-CD86, and assessed by flow cytometry. Subsequently, 1×106/mL CD11c^+^ cells were stimulated with 100 ng/mL LPS for 24 h. Cells were then harvested and incubated with GolgiStop for 4 h. IL-10 and IL-12 levels were determined by intracellular staining. **C.** CM-derived TREM-2^+^DCs manifested a reduced OVA-endocytic capacity. DCs generated from NM and CM were incubated with 1 mg/mL OVA-coupled Alexa Fluor 647 on day 5 of culture, then stained with CD11c-FITC and TREM-2-PE and analyzed by flow cytometry. OVA expression was determined by percentage and MFI on CD11c^+^TREM-2^+^ gated cells. Mean ± SEM of triplicate wells from one representative experiment out of three was shown. n=6/group in A. *p < 0.05, **p < 0.01, compared with normal mice in A; *p < 0.05 compared with NM in B and C.

TREM-2^+^DCs from the lung of tumor-bearing mice present an MHC II^low^ CD80^low^ CD86^low^phenotype (Figure 5A). Also, we obtained similar results in vitro showing impaired maturation of TREM-2^+^DCs from CM with lower levels of CD80, CD86, and MHC II I-A/I-E (Figure 5B). Furthermore, TREM-2^+^DCs from tumor-bearing mice and CM produced less IL-12 but more IL-10 (Figure 5A, 5B), In addition, we also collected DCs from CM on day 5 and tested its OVA uptake to investigate their endocytosis phenomenon. As expected, TREM-2^+^DCs from CM manifested a reduced endocytic capacity in contrast to that from NM (Figure 5C).

### CM-derived TREM-2^+^DCs performed a more potent inhibitory effect on T cells proliferation

**Figure 6:**
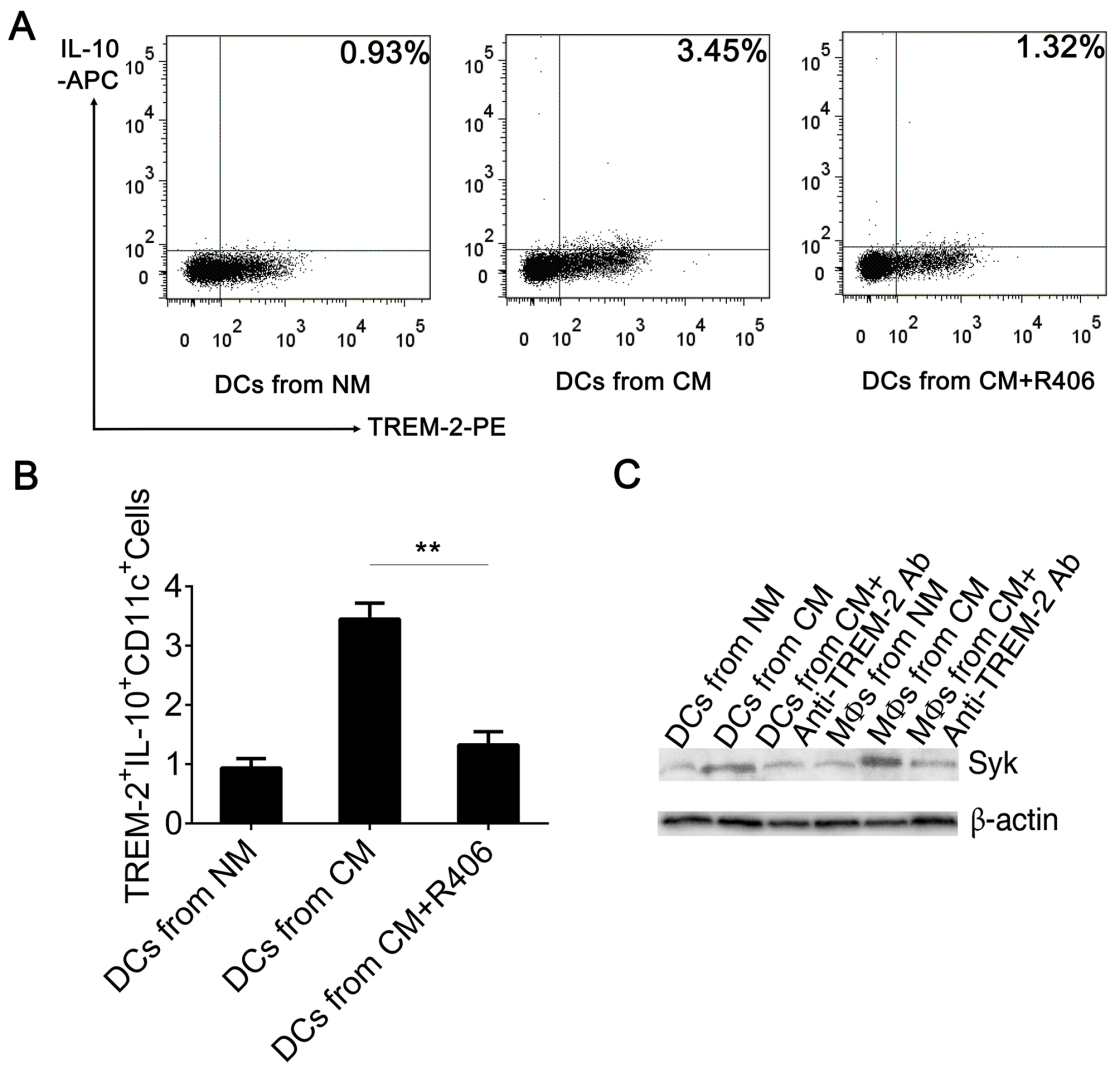
CM-derived TREM-2^+^DCs exerted a more potent inhibitory effect on T cells proliferation **A, B.** Single cell suspension of splenocytes from C57BL/6J mice were prepared for T cells negative selection. Subsequently, TREM-2^+^DCs, and TREM-2^−^DCs derived from CM were sorted and co-cultured with CFSE-staining at a ratio of 1:5. An anti-TREM-2 mAb (10 μg/mL), an IL-10 neutralizing mAb (1 ng/mL) and Syk inhibitor (R406, 4μg/mL) were added to the co-culture system, and 72 h later, proliferation of T cells was detected by FACS and analyzed by FlowJo. The program calculated the percentage of divided cells (% divided) based on the CFSE dilution levels in dividing cells. **p<0.01 compared with other four groups.

FACS analysis displayed that the proliferation of T cells was significantly inhibited in the presence of TREM-2^+^DCs with a lower frequency of dividing cells. Interestingly, TREM-2^+^DCs performed a more potent inhibitory effect on T cells proliferation in comparison with TREM-2^−^DCs (*p* < 0.05) (Figure 6A, 6B). During the co-culture of DCs and T cells as described above, addition of anti-TREM-2 mAb (10 μg/mL) to block TREM-2 on DCs was able to recover the T cell proliferation from the inhibition of TREM-2^+^DCs (Figure 6A, 6B).

### Syk is responsible for TREM-2-mediated signaling in an IL-10 dependent manner

**Figure 7:**
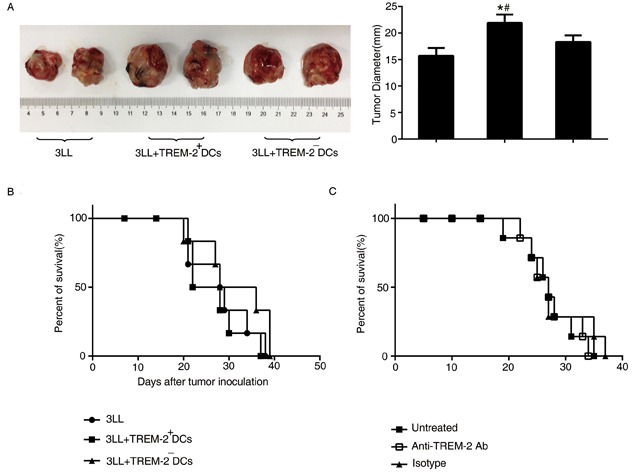
Blockage of Syk inhibited IL-10 production of TREM-2^+^DC **A, B.** Syk inhibitor (R406, 4 μg/mL) were added to CM from day 3 to day 9, following which the DCs were harvested and the CD11c^+^ population was enriched by MACS, stained with TREM-2^−^PE and IL-10-APC, and analyzed by flow cytometry. Mean ± SEM of triplicate wells from one representative experiment out of three was shown. **p < 0.01 compared with DCs from CM+R406. **C.** TREM-2 exerted negative immunoregulation through Syk tyrosine kinase. Western blot was performed with 50 μg protein extracts from DCs and MΦs per lane and probed with Syk and P-Syk Ab. Data were shown without P-Syk expression.

Several studies have discussed the intracellular signaling events induced by the activation of the TREM-2/DAP12 pathway. Upon TREM-2 binding, the ITAM tyrosines in DAP12 are phosphorylated by SRC-family kinases (SFKs) allowing the recruitment and activation of Syk kinase [11]. Here, we observed that Syk was increased in DCs and MΦs harvested from CM, which could be down-regulated by pre-exposing to anti-TREM-2-mAb while p-Syk could not be detected by western blot (Figure 7C). On the other hand, addition of R406, a Syk inhibitor, to CM from day 3 to day 9 during DC culture, demonstrated that blockade of Syk obviously inhibited IL-10 production of TREM-2^+^DC (Figure 7A, 7B). Remarkably, IL-10 neutralizing antibody and R406, both lowered the suppressive potential of TREM-2^+^DCs on the proliferation of T cells (Figure 6A, 6B).

### Adoptive transfer of TREM-2^+^DCs accelerate tumor growth rather than jeopardize the survival in lung cancer-bearing mice

**Figure 8:**
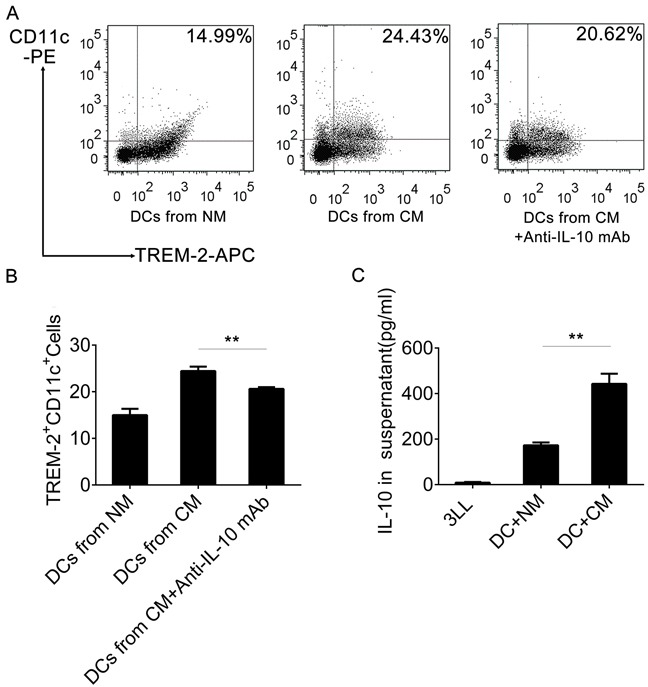
The effect of TREM-2 on tumor growth and survival **A.** Tumors were excised from subcutaneous tumor models and diameters were measured by a caliper. *p < 0.05 compared with control; #*p* < 0.05 compared with 3LL+TREM-2^−^DC group. **B.** TREM-2^+^DCs had no effect on survival of orthotopic tumor models. **C.** In vivo depletion of TREM-2 could not prolong the survival of tumor-bearing mice. The number of mice was indicated as a percentage of total mice (n = 6/group). Data are representative of three independent experiments with similar results.

3LL cells mixed with TREM-2^+^DCs and TREM-2^−^DCs (ratio = 5:1) were injected subcutaneously and the tumor diameters evaluated 28 days later. As shown in Figure 8A, TREM-2^+^DCs could accelerate tumor growth of lung cancer-bearing mice, but did not exhibit any effect on the survival of the orthotopic tumor models (Figure 8B).

Simultaneously, administration of anti-TREM-2 mAb in the lung cancer-bearing mice 7 days after tumor transplantation, depleting in vivo TREM-2, also, could not prolong the survival of tumor-bearing mice (Figure 8C).

### Blockage of IL-10 reduced TREM-2^+^DCs in CM

**Figure 9:**
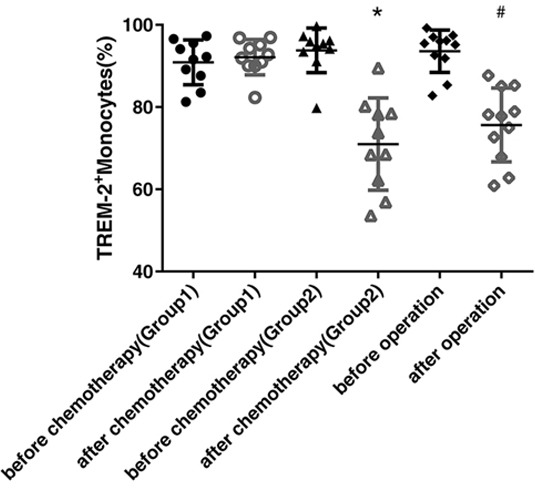
IL-10 induced TREM-2^+^DCs in CM **A, B.** An anti-IL-10 mAb (1 ng/mL) was added in the CM in on day 3 of culture and DCs harvested on day 9, stained with CD11c-PE and TREM-2^−^APC and analyzed by flow cytometry. **C.** IL-10 Levels of supernatant from 3LL, DCs+NM, and DCs+CM (day 9) were detected by ELISA. Mean ± SEM from triplicate wells of one representative experiment out of three was shown. **p < 0.01 compared with DCs from CM in (B) and compared with DCs+NM in (C).

We had already observed that TREM-2^+^DCs from CM had a high level of IL-10 production, so we assessed IL-10 levels by ELISA in the supernatant of 3LL cells, DCs, and DCs with CM, respectively. Interestingly, the IL-10 level was barely detectable in 3LL supernatant and low in DC supernatant, but extremely high in the supernatant of DCs cultured with CM (Figure 9C). Moreover, a neutralizing IL-10 mAb reduced the TREM-2^+^DCs derived from CM (Figure 9A, 9B).

## DISCUSSION

TREM-2, an important regulator in innate and adaptive immunity, has attracted so much attention since its discovery in 2001 [12]. Recent studies found that TREM-2 paired with DAP12 had a critical role in the negative regulation of DC and MΦ activation upon TLR ligation [9, 10, 13, 14]. Here, we observed that more TREM-2^+^ DCs and TREM-2^+^MΦs were induced by the tumor microenvironment. Such TREM-2^+^DCs not only presented impaired phenotypes and reduced OVA-endocytic capacity but also exercised a more potent inhibitory effect on the proliferation of T cells, suggesting that TREM-2 may exert a negative regulation in tumor immunity.

Hitherto, TREM-2 was considered to be expressed on immature monocyte derived DC, newly recruited peritoneal and alveolar MΦs, and BM-derived MΦs [10, 12, 14], but not on circulating monocytes or tissue-resident MΦs [10]. Surprisingly, we found that TREM-2 could be detected on the monocytes of peripheral blood from both, patients with lung cancer and healthy controls, also on the MΦs of lung tissues. This indicated that the expression of TREM-2 significantly increased in lung cancer patients compared to that of the healthy controls, and that in pulmonary MΦs had a positive correlation with pathological TNM staging of lung cancer. We speculated that tumor microenvironment may promote monocytes to differentiate into MΦs and induce TREM-2 expression. More importantly, efficient therapy obviously reduced its expression, implying that TREM-2 may be a valuable marker for assessment of the efficacy of lung cancer therapy. Concurrently, we also observed that the percentage of TREM-2^+^CD11c^+^DCs markedly increased in the lung tissue of orthotopic 3LL tumor model, and more TREM-2^+^DCs and TREM-2^+^MΦs could be derived from CM added with the 3LL supernatant. Taken together, these results speculated that although TREM-2 had a protective role in maintaining homeostasis of osteoclast and CNS development [15, 16], it might be more likely to be involved in the negative modulation of immune function and facilitate immune evasion in the tumor bearing host.

Ito et al. reported that after stimulation with TLR ligands, IL-12 and type I IFN production from TREM-2-deficient DCs was notably increased over wild-type (WT) DCs while TNF and IL-6 secretions were also modestly higher. In addition, TREM-2-deficient BMDCs had increased TLR-induced maturation and were more efficient in inducing T-cell proliferation exposed to TLR ligand as compared to the WT BMDCs [9]. These facts suggested that TREM-2 negatively regulated TLR responses in DCs. DCs isolated from the tumor-bearing host, appearing to be phenotypically and functionally defective, can assist the tumor to escape immune surveillance [17, 18]. Turnbull and co-workers proved that alternative activation of resident peritoneal MΦs induced by IL-4 and IL-13 led to TREM-2 expression, whereas IFN-γ and LPS rapidly abrogated it. TREM-2 functioned to inhibit TNF-α and IL-6 production by MΦs in response to TLR ligands [10], whereas overexpression in myeloid cells led to less TNF and inducible nitric oxide [19], but more IL-10 [19, 20]. According to the current opinion, MΦs have been classified into M1 and M2. M1 are immune effector cells activated by LPS and IFN-γ. In contrast, M2 are induced by IL-4 or IL-13, display impaired antigen presenting capacity, and turn off immune system activation [21]. TAMs generally acquire an M2-like phenotype, presenting high levels of IL-10 and low levels of IL-12 that play a crucial role in tumor progression [22].

In our study, we found that MHCII I-A/I-E, CD80, and CD86 were down-regulated in TREM-2^+^CD11c^+^DCs from tumor-bearing mice. After LPS stimulation, such DCs produced less IL-12 but more IL-10. Although, similar results were obtained in vitro, the phenotype of tumor DCs was controversial. Gottfried's study indicated that the phenotype of DCs in tumor tissue may depend on the specific tumor type [23]. Perrot et al. detected that in most non-small lung cancer cells, tumor-infiltrating DCs were immature and expressed none of the activation markers, CD80 or CD86 [17], which was consistent with our findings that TREM-2^+^DCs from CM and tumor-bearing mice presented CD80^Low^CD86^Low^MHCII^Low^ phenotypes.

It is generally recognized that T cells are critical in inhibiting tumor cells. Tumor-induced DCs could inhibit the proliferation of T cells directly through immunosuppressive cytokines such as IL-10 or TGF-β [24]. Schneider et al. reported that the lung cancer-derived DCs, with elevated secretion of IL-10, significantly reduced the levels of T cell proliferation [25]. Herein, we also observed CM-derived TREM-2^+^ DCs performed a more potent inhibitory effect on T cells proliferation while blockade of TREM-2 partially restored the proliferation. The inhibition of TREM-2/DAP12 signaling pathway requires the ITAM tyrosines and presumably involves Syk, as the Syk-deficient BM-derived MΦs also exhibit enhanced cytokine production in response to TLR-induced activation, which is in line with DAP-12 deficient MΦs [14]. Furthermore, we also found that the elevation of TREM-2 was coupled with Syk recruitment, and that its blockage could attenuate the expression of Syk. Moreover, inhibition of Syk could reduce IL-10 secretion of TREM-2^+^DCs and lower the suppression potential of TREM-2^+^DCs on T cell proliferation. Thus we speculated that TREM-2 might exert negative regulation through Syk pathway in an IL-10 dependent manner. Syk recruitment and activation evoked downstream signaling cascades, including phospholipase C-γ activation induced calcium flux and Ras activation mediated extracellular signal-regulated kinase (ERK) activation, which need to be investigated in the future.

Additionally, we observed that tumor cells secreted little IL-10, but tumor- containing medium induced a strong IL-10 production by DCs. Importantly, a neutralizing IL-10 mAb reduced TREM-2^+^DCs derived from CM and reversed the inhibitory effect of TREM-2^+^DCs in T cell proliferation. These results indicated that IL-10 may not only induce TREM-2^+^DCs in the tumor microenvironment but also be the effective factor of TREM-2^+^DCs during the negative regulation of the tumor. A feedback loop may be existent between TREM-2 and IL-10.

In conclusion, we have shown that TREM-2 might act as a negative immuno-regulatory molecule through Syk pathway in an IL-10 dependent manner, and partially predict prognosis in patients with lung cancer. Our study provides a new mechanistic insight into tumor immune evasion.

## MATERIALS AND METHODS

### Mice, cell lines, and reagents

C57BL/6 mice were purchased from SLAC laboratory Animal Co., Ltd. (Shanghai, China) and kept in Laboratory Animal Center in the first affiliated hospital of Zhejiang University hospital. Mice aged 6-8 weeks were used. This study was performed with the approval of The local Animal Care and Use Committee and all the experiments were performed according to the National Institutes of Health Guide for the Care and Use of Laboratory Animals. The Lewis lung carcinoma cell line (3LL) was obtained from American Type Culture Collection (ATCC) and maintained in RPMI 1640 medium and 10% (v/v) heat-inactivated fetal bovine serum (FBS) (PAA Laboratories; Dartmouth, MA, USA). Recombinant mouse granulocyte-macrophage colony stimulating factor (GM-CSF) and recombinant mouse IL-4 were obtained from PeproTech (Rocky Hill, NJ, USA). Mouse T Cell Enrichment Kit and Mouse PE Positive Selection Kit were obtained from Stem Cell technologies (Vancouver, BC, Canada). Lipopolysaccharide (LPS), penicillin, streptomycin, Alexa Fluor 647-labeled OVA were obtained from Sigma-Aldrich (St. Louis, MO, USA). The CellTrace CFSE Cell Proliferation Kit and mouse T-activator CD3/CD28 for T cell expansion and activation was obtained from Invitrogen (Carlsbad, CA, USA). Human TREM-2 polyclonal antibody (goat IgG) and human F4/80 polyclonal antibody (rabbit IgG) were purchased from R&D system (Minneapolis, MN, USA) for immunohistochemistry. Anti-Syk antibody and Anti-Syk (phospho Y323) antibodies were obtained from Abcam (Cambridge, UK). Rat anti-mouse TREM-2 monoclonal antibody (clone 78.18) was obtained from Genway Biotech (San Diego, CA, USA) for TREM-2 blocking, R406 (Syk inhibitor) from Selleckchem (Houston, TX, USA) for Syk blockage and mouse IL-10 antibody were obtained from R&D Systems for neutralization. The antibodies for flow cytometry including APC-labeled anti-mouse CD4, IL-10 and IL-12, FITC-labeled anti-mouse CD11c, PerCP-Cy5.5 labeled anti-mouse CD80, and the respective isotype controls were obtained from BD Pharmingen (San Jose, CA, USA). PE-labeled anti-mouse/anti-human TREM-2 and APC-labeled anti-mouse/human TREM-2 were from R&D system; PE-labeled anti-mouse CD14, PerCP-eFluor710 labeled anti-mouse MHCII I-A/I-E, and PE-labeled anti-mouse F4/80 were from eBioscience (San Diego, CA, USA); PerCP-labeled anti-mouse CD86 was from Biolegend (San Diego, CA, USA). Lysis buffer and GolgiStop were from BD Pharmingen.

### Subjects and blood sample preparation

52 patients (33 males and 18 females, age range from 41 to 81 years) with a histologically confirmed diagnosis of primary lung cancer and 41 healthy controls (27males and 14 females, age range from 35 to 78 years) were recruited in the first affiliated hospital of Zhejiang University hospital between April 2012 to July 2012. Patients that received any therapy previously were excluded. 5 mL of peripheral blood from patients and healthy controls, anticoagulated with EDTA, was used for the isolation of peripheral blood mononuclear cells (PBMCs) that were analyzed by flow cytometry. Also, we collected blood samples from an additional 20 patients with lung cancer before and after chemotherapy (at the beginning of cycle 3) at the department of respiratory disease, and 15 patients with lung cancer before and after one month of operation at the department of thoracic surgery, from September 2012 to June 2013. Therapeutic responses of patients were assessed according to Response Evaluation Criteria In Solid Tumors (RECIST, version1.1) [26]. PR (Partial Response); PD (Progressive Disease); SD (Stable Disease). The study was approved by the local Ethics Committee, and informed consents were obtained from each patient or representative.

### Orthotopic and subcutaneous tumor models

Orthotopic lung cancer model was prepared by intrapulmonary inoculation with 3LL cells as previously described [27]. Briefly, C57BL/6 mice were anesthetized with 1% (v/v) pentobarbital sodium. A 5 mm skin incision to the right chest was made and the muscle was separated from costal bones. After observing the lung motion through the pleura, a 28-gauge needle attached to a 100 μL microsyringe was directly inserted through the 6th intercostal space into the lung to a depth of 3mm. 3LL cells (2×10^5^ per mouse) suspended in 40 μL of PBS containing matrigel (v/v= 1/1), were injected into the lung parenchyma. In order to block the effect of TREM-2, 50 μg anti-mouse TREM-2 monoclonal antibody or its isotype was administrated via intraperitoneal injection on day 7.

Parallely, 1×10^5^ 3LL tumor cells, in 0.1 mL PBS as a single-cell suspension was injected subcutaneously on the quadriceps of the left leg. Animals were examined three times each week and 28 days later, entire tumors were removed and diameters measured with a caliper.

### Isolation and culture of BM-derived DCs and BM-derived MΦs

Mice were sacrifice by CO_2_ Asphyxiation. Femurs and tibias were removed and mechanically purified from surrounding tissues with epiphyses. 25-G needle syringe was filled with 1mL cold sterile PBS and inserted into BM cavity and flushed with 1mL of the wash medium 4-5 times until the cavity turned white. The wash medium was collected and centrifuged at 500×g for 10min to remove the supernatant. The cells were gently resuspended in 3 mL erythrocyte lysis buffer. The pellet with PBS. For DC generation, BM cells were cultured in a 6-well plate at an initial density of 1×10^6^/ml in 3 mL RPMI 1640 with 100 U/mL penicillin, 100 μg/mL streptomycin, 10% FBS, 10 ng/mL GM-CSF and 1 ng/mL IL-4 [28]. After 48 h, non-adhering cells were gently washed out and the culture continued for 5-7 days. For MΦ generation, BM cells were cultured as above at an initial density of 5×10^5^/ml in RPMI 1640 with 100 U/ml penicillin, 100 μg/ml streptomycin, 10% FBS and 10^4^ U/ml M-CSF [29] with 3 mL additional medium on day 3. The digested MΦs were collected after 7 days. During culture, we prepared CM containing supernatant of 3LL cells (medium: supernatant=4:1) mimicking tumor microenvironment for DC and MΦ derivation, which was added to the DC medium from day 3 until day 9, and to the MΦ medium from day 3 to day 7.

### Isolation and purification of T cells and TREM-2^+^DCs

Single cell suspension of splenocytes from normal or tumor-bearing mice were prepared. T cells were negatively selected with T Cell Enrichment Kit according to the manufacturer's instructions. The purity of T cells (CD3^+^cells) was >90%. BM- derived DCs were harvested from CM on day 9, then CD11c^+^TREM-2^+^DCs and CD11c^+^TREM-2^−^DCs were sorted by high-speed cell sorter (BD FACSAria III), the purity of which was confirmed to be ≥95%.

### Flow cytometry analysis

PE-labeled anti-human TREM-2 was used for the detection of TREM-2 expression on monocytes in peripheral blood from patients. PE-labeled anti-mouse CD14 and APC-labeled anti-mouse TREM-2 was used to detect the TREM-2 expression on monocytes in peripheral blood from mice. CD11c^+^ cells were isolated from lungs of tumor-bearing and normal mice by magnetic activated cell sorting (MACS), incubated for 15 min on ice with Fc block (anti-CD16, anti-CD32) (BD Biosciences) and stained for an additional 30 min at room temperature with the following antibodies: PerCP-eFluor 710 labeled anti-mouse MHC II I-A/I-E, PerCP-Cy 5.5 labeled anti-mouse CD80, PerCP labeled anti-mouse CD86, and PE labeled anti-mouse TREM-2. For intracellular staining, CD11c^+^ cells isolated from murine lungs were stimulated with 100 ng/mL LPS for 24 h and incubated with GolgiStop for 4 h. After which the cells were surface stained for TREM-2, fixed and permeabilized with IC Fixation/Permeabilization buffer (eBioscience), washed, and intracellularly stained with APC-labeled anti-mouse IL-12 and IL-10. Flow cytometric acquisition was performed using an FACS Calibur (BD Biosciences), and data were analyzed by CellQuest software (BD Biosciences).

### Endocytosis of soluble OVA

Purified DCs generated as described above were incubated with 1 mg/mL OVA-coupled Alexa Fluor 647 for 45 min at 37°C on day 5 of culture [30] followed by staining with FITC labeled anti-CD11c and PE-labeled anti-TREM-2 for analysis by flow cytometry.

### Monitoring T cell proliferation with carboxyfluorescein diacetate succinimidyl ester(CFSE)

T cell proliferation assay was developed by CFSE Cell Proliferation Kit based on the manufacturer's protocol. Briefly, T cells were resuspended in pre-warmed PBS-0.1% BSA at a final concentration of 1×10^6^ cells/mL. The cells were incubated with 10 μM CFSE solution at 37°C for 10 min protected from light. The staining was quenched by the addition of 5 volumes of ice-cold RPMI 1640 with a further 5 min incubation on ice, following which the cells were washed and cultured under different conditions in 96-well plates. After 72 h, T cells were harvested, stained for APC labeled anti-mouse CD4, and detected by flow cytometry. The results were analyzed by FlowJo software (Treestar Inc., Ashland, OR, USA). The program calculated the percentage of divided cells (% divided) based on the CFSE dilution in dividing cells [31, 32]

### Immunohistochemistry analysis

71 surgical specimens from patients with lung cancer and 21 surgical specimens from patients with benign lung diseases were collected for immunohistochemistry. TNM staging was determined according to the 7th TNM classification [33]. Paraffin-embedded lung sections (4μm) were immunostained with TREM-2 polyclonal antibody to evaluate its the expression on MΦs around the tumor cells; MΦs were identified by F4/80 polyclonal antibody. The staining area was quantified using a light microscope (DP20, Olympus, Melville, NY) and the attached image analysis system (Image-Pro Plus 5.1). The intensity of TREM-2 staining was quantified using mean integrated optical density (IOD) (sum IOD/sum area) [34].

### Western blot analysis

DCs and MΦs were harvested for protein extraction. Protein electrophoresis was performed using SDS-PAGE (10% SDS tricine gel) and transfer to polyvinylidene difluoride membrane. The membranes were blocked and immunoblotted with anti-Syk mAb (1:1000; Santa Cruz Biotechnology, Dallas, TX) or anti-P-Syk mAb overnight at 4°C followed by HRP-conjugated secondary Ab (1:5000; Santa Cruz Biotechnology). The membranes were washed extensively and the immunoreactions were visualized by ECL kit (Thermo Scientific, Waltham, MA) based on the manufacturer's instructions.

### Statistical analysis

Results were expressed as mean ± SEM and analyzed by ANOVA (Tukey honest significant difference), or independent t-test as appropriate with SPSS 21.0 (SPSS, Chicago, IL). Statistical significance was accepted at *p* < 0.05.

## SUPPLEMENTARY FIGURE


