# Supplementary material for: TREM-2 serves as a negative immune regulator through Syk pathway in an IL-10 dependent manner in lung cancer
Source: Oncotarget. 2016 Apr 18;7(20):29620–34. doi: 10.18632/oncotarget.8813 (PMC5045421; doi:10.18632/oncotarget.8813)
Supplement: Supplementary file 1 [file oncotarget-07-29620-s001.pdf]

SUPPLEMENTARY FIGURE

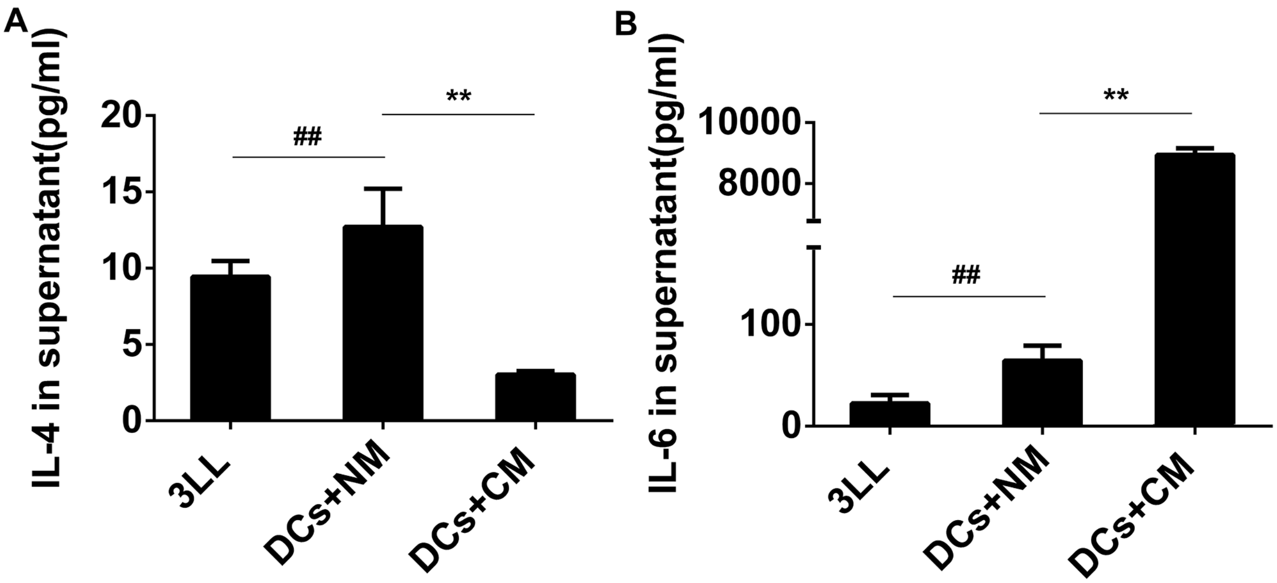

Supplementary Figure S1: The levels IL-4 A. and IL-6 **B.** of supernatant from 3LL, DCs+NM and DCs+CM (day 9) were detected by ELISA. Mean ± SEM of triplicate wells from one representative experiment out of three was shown. ## $p < 0.01$ compared with 3LL groups, **$p < 0.01$ compared with DCs+NM.
